# The common HLA class I-restricted tumor-infiltrating T cell response in HPV16-induced cancer

**DOI:** 10.1007/s00262-022-03350-x

**Published:** 2022-12-16

**Authors:** Saskia J. Santegoets, Marij J. P. Welters, Deborah S. Schrikkema, Manon R. Freriks, Hanna Kok, Bianca Weissbrich, Anouk van den Branden, Carsten Linnemann, Ton N. Schumacher, Sabina Adhikary, Gavin Bendle, Sjoerd H. van der Burg

**Affiliations:** 1 Department of Medical Oncology, Oncode Institute, Leiden University Medical Center, Building 1, C7-P, PO box 9600, 2300 RC Leiden, The Netherlands; 2 Kite, a Gilead Company, Amsterdam, The Netherlands; 3 Division of Molecular Oncology and Immunology, Oncode Institute, The Netherlands Cancer Institute, Amsterdam, The Netherlands; 4 Department of Hematology, Leiden University Medical Center, Leiden, The Netherlands; 5 Kite, a Gilead Company, Santa Monica, CA USA

**Keywords:** HPV16, TCR gene transfer, TIL

## Abstract

Immunotherapies targeting truly tumor-specific targets focus on the expansion and activation of T cells against neoantigens or oncogenic viruses. One target is the human papilloma virus type 16 (HPV16), responsible for several anogenital cancers and oropharyngeal carcinomas. Spontaneous and vaccine-induced HPV-specific T cells have been associated with better clinical outcome. However, the epitopes and restriction elements to which these T cells respond remained elusive. To identify CD8^+^ T cell epitopes in cultures of tumor infiltrating lymphocytes, we here used multimers and/or a functional screening platform exploiting single HLA class I allele-engineered antigen presenting cells. This resulted in the detection of 20 CD8^+^ T cell responses to 11 different endogenously processed HLA-peptide combinations within 12 HPV16-induced tumors. Specific HLA-peptide combinations dominated the response in patients expressing these HLA alleles. T cell receptors (TCRs) reactive to seven different HLA class I-restricted peptides could be isolated and analysis revealed tumor reactivity for five of the six TCRs analyzed. The tumor reactive TCRs to these dominant HLA class I peptide combinations can potentially be used to engineer tumor-specific T cells for adoptive cell transfer approaches to treat HPV16-induced cancers.

**Supplementary Information:**

The online version contains supplementary material available at 10.1007/s00262-022-03350-x.

## Background

Human papilloma virus (HPV)-induced cancers of the cervix, penis, anus, vulva, vagina and oropharynx represent 4.8% of all cancers worldwide [1]. HPV16 is the most dominant HPV type detected in cancers at all anatomical sites [2, 3]. These tumors express the viral oncoproteins E6 and E7, which serve as target antigens for both CD4^+^ and CD8^+^ tumor-infiltrating T cells (TILs) [4–6]. Several lines of evidence make a strong case for their role in cancer control. Regression of vulvar lesions [7, 8] or superior clinical benefit in oropharyngeal cancer [5] has been related to a strong spontaneous presence of HPV16-specific type 1 T cells. Furthermore, therapeutic vaccines that significantly reinforced the E6- and E7-specific T cell response induced partial and complete regressions of HPV-induced high-grade precursor lesions of the cervix and vulva [9, 10] and, in combination with PD-1 blockade, showed clinical efficacy in incurable HPV16-induced oropharyngeal cancer [11]. In addition, protocols that use HPV-specific T cells obtained from TILs [12], tumor-draining lymph nodes [13] or after in vitro TCR engineering [14] are currently being explored for adoptive T cell therapy.

The clinical evidence of the antitumor effects of HPV-specific T cells has renewed the interest in the identification of clinically relevant CD8^+^ T cell epitopes and their HLA-restriction elements. Clinically relevant epitopes are defined as those that are recognized by CD8^+^ TILs when presented on tumor cells. However, proper tumor recognition does not always occur since HPV-induced cervical and oropharyngeal cancer cells can evade recognition due to low expression of the transporter associated with antigen processing (TAP), the proteasome subunits and/or low cell surface HLA class I expression [15, 16]. Despite extensive HLA class I-restricted epitope identification studies in the past, the evidence that these epitopes are recognized on tumor cells is limited. To our knowledge, only six of such epitopes have been identified since 1997 [6, 14, 17, 18], one for which the presentation has also been confirmed by mass spectrometry [19].

We previously described that part of the HPV16-induced cervical carcinomas (CxCa) and the majority of oropharyngeal squamous cell carcinomas (OPSCC) are infiltrated by both CD4^+^ and CD8^+^ HPV16-specific T cells [4, 5]. In OPSCC their presence is strongly related to better overall survival [5, 20]. Here, we used multimers and/or a functional screening platform exploiting antigen presenting cells engineered to express a single defined common HLA class I allele to identify CD8^+^ T cell epitopes in TIL cultures obtained from 16 patients with HPV16-induced cancer. A total of 20 CD8^+^ T cells responses were detected within 12 patients, which were directed against 11 different endogenously processed HLA-peptide combinations. T cell receptors (TCRs) reactive to seven different HLA class I-restricted peptides could be isolated and tumor reactivity was confirmed for five of the six TCRs analyzed. The isolated tumor reactive TCRs to these dominant HLA class I peptide combinations potentially can be used to engineer tumor-specific T cells for adoptive cell transfer approaches in patients with HPV16-induced cancers.

## Materials and methods

### Sample

#### Patients

Patients included in this study were part of two larger observational studies on CxCa and OPSCC. Women with histologically proven CxCa (International Federation of Gynecology and Obstetrics 1a2, 1b1/2) were included in the CIRCLE study (P08–197) investigating cellular immunity against anogenital lesions [4]. Patients with histology-confirmed OPSCC were included in the P07–112 study investigating the circulating and local immune response in patients with head and neck cancer [5]. Patients were included after signing informed consent. Both studies were conducted in accordance with the declaration of Helsinki and approved by the local medical ethical committee of the Leiden University Medical Center (LUMC) and in agreement with the Dutch law. HPV typing and p16^ink4a^ immunohistochemistry (IHC) staining was performed on formalin-fixed paraffin-embedded (FFPE) tumor sections at the LUMC Department of Pathology as described [5]. HLA class I type of the patients was determined by PCR using sequence-specific oligonucleotides as described previously [21].

#### TIL culturing

CxCa and OPSCC tumors were obtained and handled as described previously [5, 20]. In brief, tumor material was cut into small pieces and the pieces were incubated for 60 min at 37 °C in Iscove’s Modified Dulbecco’s Medium (IMDM, Lonza, Verviers, Belgium) with 10% human AB serum (Capricorn Scientific, Esdorfergrund, Germany) and supplemented with high dose of antibiotics (50 µg/ml Gentamycin (Gibco/Thermo Fisher Scientific (TFS), Bleiswijk, the Netherlands), 25 µg/ml Fungizone (Invitrogen/TFS), 100 IU/ml penicillin (pen; Gibco/TFS) and 100 µg/ml streptomycin (strep; Gibco/TFS)). Next, the tumor pieces were put in culture in IMDM supplemented with 10% human AB serum, 100 IU/ml penicillin, 100 µg/ml streptomycin, 2 mM L-glutamin (Lonza, Breda, Netherlands; hereafter referred to as IMDM complete) and 1000 IU/ml human recombinant IL-2 (Aldesleukin, Novartis, Arnhem, the Netherlands). Cultures were replenished every 2–3 days with fresh IMDM complete and IL-2 to a final concentration of 1000 IU/ml and when sufficient T cells were obtained after 2–4 weeks the cells were harvested, cryopreserved and stored in liquid nitrogen until use.

#### HPV16-specific T cell reactivity analysis

To determine the specificity of T cells infiltrating the tumor, TIL batches from HPV16^+^ CxCa and OPSCC tumors were analyzed for the presence of HPV16-specific T cells using the 5 days [3H]-thymidine-based proliferation assay as described [5, 20]. In brief, T cells were tested in triplicate against autologous HPV16 E6/E7 synthetic long peptide (SLP; 22-mers with 14 amino acids overlap) loaded autologous monocytes. PHA (0.5 µg/ml; HA16 Remel; ThermoFischer Scientific) was taken along as positive control, while unloaded monocytes served as negative control. At day 1.5 and 4 supernatant (50 µl/well) was harvested to determine cytokine production. During the last 16 h of culture, 0.5 μCi/well of [3H]thymidine was added to measure proliferation. The stimulation index was calculated as the average of test wells divided by the average of the medium control wells. A positive response was defined as a stimulation index of at least 3. Antigen-specific cytokine production was determined by cytometric bead array (CBA, Th1/Th2 kit, BD Bioscience, Breda, the Netherlands) according to the manufacturer’s instructions. The cutoff value for cytokine production was 20 pg/ml, except for IFNγ for which it was 100 pg/ml. Positive cytokine production was defined as at least twice above that of the unstimulated cells.

### CD8^+^ T cell identification platform

**Fig. 1:**
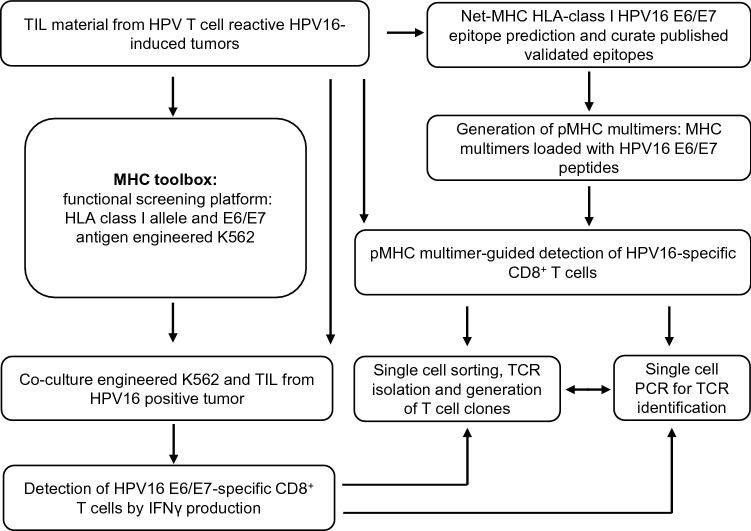
Experimental set-up for the detection and isolation of HPV16-specific CD8^+^ T cells. Pipelines for the detection and isolation of CD8^+^ T cells from tumor infiltrated lymphocytes (TILs) through pMHC class I multimer staining and the functional screening platform. TIL batches were obtained from HPV16 positive tumors by culturing tumor fragments with 1000 U/ml IL-2. HPV16 E6 and E7 epitopes presented in common and relevant HLA class I were predicted using the Net-MHC platform. The obtained predicted epitopes were curated from published validated ones and used to generate pMHC class I multimers for the detection of HPV16 E6/E7-specific CD8^+^ T cells. In parallel, K562 engineered to express single defined HLA class I and target antigen (shuffled E6 (E6sh) or E7 (E7sh)) were generated and used to screen TIL batches for HPV-specific CD8^+^ T cells. Detection and single cell sorting of HPV-specific CD8^+^ T cells was done following co-culture by making use of the flow cytometry-based IFNγ-secretion assay. Single cell sorted HPV-specific CD8^+^ T cells were used to generate T cell clones and single cell PCR was applied to identify the TCR

In order to identify and develop TCRs for HPV16 E6 and E7 epitopes that are restricted to common HLA-class I alleles, TIL batches of HPV16^+^ T cell reactive tumors were screened for the presence of HPV16 E6 and E7-specific CD8^+^ T cells using a major histocompatibility complex (MHC) toolbox based on either the peptide-MHC (pMHC) multimer technology or an HLA class I functional screening platform (Fig. 1).

#### Detection of HLA class I restricted TCR through pMHC multimer staining

Putative HPV16 E6 and E7 epitopes of 9, 10 or 11 amino acids were either published and validated epitopes or identified by affinity-based algorithmic prediction using NetMHC3.4 for the highly prevalent HLA class I alleles (see Supplemental Table 1) [22]. pMHC class I multimers were generated using conditional MHC class I ligands and peptide exchange technology and used for combinatorial-encoded multimer staining as described [23]. In brief, to enable the simultaneous detection of two-color combinations of multiple epitopes in one sample, each MHC class I multimer was labelled with two different streptavidin conjugated fluorochromes. TIL batches were stained with 7AAD (Invitrogen) or LIVE/DEAD Fixable Near-IR dead cell stain kit (Thermofisher) according to manufacturer’s instructions. Next, cells were stained by 1 μl of PE-labelled multimers, 2 μl of allophycocyanin (APC)-, BB515- or BV650-labelled multimers or 3 μl of PeCy7- or BV421-labelled multimers in 50 μl of PBS + 5% HS for 15 min at 37 °C. Following washing, surface antibody staining was performed as normal by exposing cells to pre-determined concentrations of CD8 antibody for 30 min at 4 °C. T cells were washed twice with PBS + 5% HS, resuspended in 200 μl of PBS + 5% HS and acquired on an BD LSRII, and analyzed using FlowJo software. A list of the tested MHC/peptide combinations is given in Supplemental Table 2.

#### Detection of HLA class I restricted TCRs through the functional screening platform

In order to detect HPV16 E6 and E7-specific T cell responses restricted to less prevalent and/or less characterized HLA class I alleles, the TIL batches were also tested against engineered K562 antigen presenting cells (APC). To this end, K562 cells, which by itself do not express HLA class I and II antigens, were engineered to express single defined HLA class I alleles and target antigens E6 or E7 (shuffled E6 and or E7 (E6sh, E7sh)), and used for the detection and isolation of HPV-specific CD8^+^ T cells. The HLA class I alleles and the artificial (shuffled) HPV16 E6 or E7 genes [24] were inserted into a retroviral pMX vector and retrovirus production and subsequent transduction was done as described [22]. Screening for HPV16-specific T cells was done by 24 h co-culture of the TIL batches with K562 target cells (ratio 3:1) in triplo, after which supernatants were harvested and assessed for IFNγ production by cytometric bead array (CBA) according to manufacturer’s instructions. Responses were considered positive when the amount of IFNγ produced by the K562-stimulated T cells was at least two times that of the unstimulated T cells (T cells alone).

### Single cell sorting and paired TCRα/β amplification and sequencing of HPV16-reactive CD8^+^ T cells

#### Single cell sorting

Single cell sorting of HPV16 E6/E7-reactive CD8^+^ T cells for TCR isolation was performed as described [25]. The cells were identified by CD8/pMHC class I multimer staining as described above or by IFNγ production using the IFNγ Secretion Assay Detection Kit (Miltenyi biotec) according to manufacturer’s instructions. In brief, sorting of CD8^+^multimer^+^ T cells from TIL batches was done directly following CD8/pMHC multimer staining and CD8^+^IFNγ^+^ T cells after overnight co-culture with K562 cells expressing the appropriate HLA class I allele and the shuffled HPV16 E6 or E7 genes. Background signal was set on irrelevant multimer or unstimulated TIL to identify true positive cells. HPV16 E6/E7-reactive CD8^+^ T cells were sorted at 1 cell/well into PCR plates containing lysis buffer. Sorted cells were either lysed immediately after sorting by incubating PCR plates in thermocyclers at 70 °C for 90 min or immediately frozen at −80 °C for later processing. Subsequent paired TCRα and β amplification, TCRα/β sequencing and data analysis were performed as described [25].

#### Retroviral transduction and expression of T cell receptors

PCR products from the single-cell PCRs were cloned into the pMP71-TCR-flex retroviral vector and the retroviral particles were generated as described [25]. Generation of the retroviral particles was performed as described [22]. In brief, Phoenix Ampho cells were plated into 10 cm dishes at 1.2 × 10e6 cells per dish, and after 24 h cells were transfected with 10 µg retroviral vector DNA using Fugene 6 transfection reagent. Viral supernatant was harvested after 48 h, added to retro-virus-coated plates (Takara), centrifuged at 2000xg at 32 °C for 2 h, and subsequently used to transduce activated human T cells from buffy coats. To this end, PBMC were incubated with CD3/CD28 human T cell expander beads (Life Technologies) for 30 min at a 1:1 ratio, after which the bead-bound CD3^+^ T cells were cultured in the presence of 100 IU/ml rhIL-2 and 5 ng/ml rhIL-15 (Peprotech). After 48 h, the activated CD3^+^ T cells were added to retrovirus-coated plates, centrifuged for 5 min at 500×*g* and incubated at 37 °C. Next day, medium was refreshed and transduction efficiency was determined after 72 h by pMHC class I multimer and/or murine TCRβ constant domain antibody staining.

#### Functional assay with TCR-transduced CD8^+^ T cells

To test the functionality of the TCR-transduced (Td) CD8^+^ T cells, the cells were analyzed by intracellular cytokine staining using flow cytometry. To this end, TCR transduced CD3^+^ T cells were incubated with target cells in triplo at a *E*:*T* ratio of 1:1 for 6 h at 37 °C in the presence of Golgiplug, after which the cells were washed and stained with antibodies against CD4, CD8 and IFNγ. Intracellular IFNγ staining was done using the cytofix/cytoperm kit according to manufacturer’s instructions. Target cells used were T2 cells or K562 cells pulsed with the cognate peptide, K562 expressing the appropriate single HLA class I allele and shE6/E7 target antigen and/or various HPV16-expressing tumor cell lines expressing the appropriate HLA class I allele (naturally or after retroviral engineering). Peptide pulsing of T2 or class I-expressing K562 cells was done for 90 min at 37 °C. Tumor cells were pre-seeded 72 h prior to the TCR reactivity assay, of which the last 48 h in the presence of 200 U/ml IFNγ. PMA and ionomycin (5 ng/ml and 500 ng/ml, respectively) stimulation was taken along as positive control. Data were normalized by correction of percentage of CD8^+^IFNγ^+^ T cells with the frequency of TCR Td CD8^+^ T cells as measured by pMHC class I multimer or mouse TCRβ chain constant domain staining on the day of the assay.

## Results

### ***Detection of 11 different HLA class I-restricted epitopes in 20 different HPV16-specific CD8***^+^***T cell responses***

In order to identify novel HPV16 E6/E7-reactive CD8^+^ T cell epitopes, TIL batches of HPV16 E6/E7-reactive tumors (Supplemental Fig. 1a) were analyzed by our HLA class I functional screening platform (Fig. 1). To this end, K562 cells expressing one of the selected common HLA class I alleles HLA-A (*n* = 5), HLA-B (*n* = 9) or HLA-C (*n* = 6) (Supplemental Table 1) and the target antigen HPV16 E6 or E7 proteins were co-cultured overnight with the TIL batches, after which HPV16-specific CD8^+^ T cell responses were detected by measuring IFNγ production. To test the sensitivity of this functional assay, we generated TCR-transduced (Td) T cells specific for the previously described HLA-A*02:01-restricted HPV16 E7_11–19_ epitope [4], which we isolated from a cervical cancer TIL culture with known specificity (Supplemental Figure 1b), spiked them into an healthy donor-derived CD8^+^ T cell fraction and tested them against K562 E7sh HLA-A*02:01 cells. Analysis showed that this allowed for the detection of HPV16-specific CD8^+^ T cells with a TCR that is able to recognize the endogenously processed epitope at frequencies above 0.01% (Supplemental Figure 2).

**Fig. 2:**
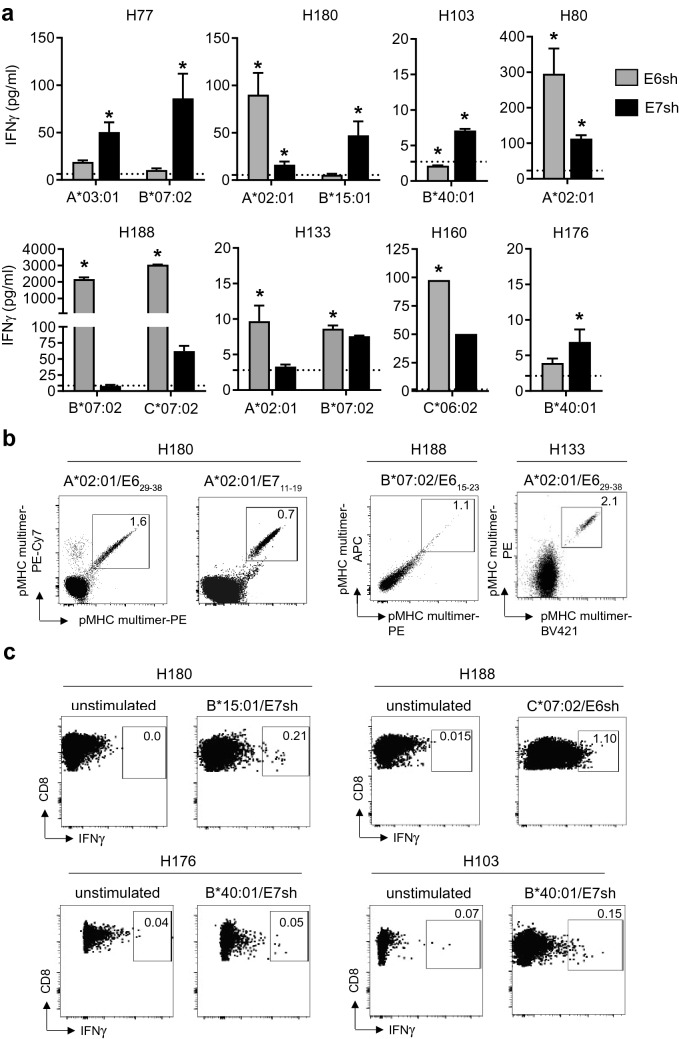
HPV16 E6 and E7-specific CD8^+^ T cells that were restricted to various HLA class I alleles could be detected with our pMHC class I multimer and/or functional screening platform. HPV16 E6- and E7-specific CD8^+^ T cells were detected in TIL batches of HPV16 positive tumors following co-culture with K562 expressing a single defined HLA class I allele and shuffled E6 (E6sh) or E7 (E7sh). **a** Graphs depicting the amount of IFNγ (pg/ml) produced in response to E6sh (grey) and E7sh (black) expressing K562 cells for eight TIL batches. Each used K562 cell batch was transfected with the depicted HLA class I allele. The asterisk depicts the detection of a positive response, which is defined as IFNγ production that is at least two times that of the T cells alone (dotted line). To confirm the presence of HPV16-specific CD8^+^ T cells in the TIL batches, pMHC class I multimer staining **b** or CD8/IFNγ staining **c** of the TIL batches was performed. **b** pMHC class I multimer double staining of the HLA-A*02:01-restricted epitopes E6_29–38_, E7_11–19_ and E6_15–23_ are depicted for three TIL batches. **c** CD8/IFNγ staining of unstimulated and K562-HLA-B*15:01/E7sh, K562-HLA-C*07:02/E6sh or K562-HLA-B*40:01/E7sh-stimulated T cells are depicted for four TIL batches

Functional screening of the HPV16 E6/E7-reactive TIL batches revealed that CD8^+^ T cell responses, able to recognize endogenously processed E6 and/or E7, could be detected among 10 of the 14 TIL tested. These responses were restricted by one or more alleles, with a total of 11 different HLA-allele:antigen combinations within the 16 responses detected (Fig. 2a, Table 1). Epitope screening using HLA-multimers (when available) resulted in the detection of 9 epitopes via HLA multimers, two which were detected in the 2 TIL populations with known HPV-reactivity and not tested in functional screen [4] and two which were found at low frequencies in TIL populations that were negative in the functional screen (Fig. 2b, Supplementary Figure 3, Table 1, Supplementary Table 3). In two patients, a response to three different HLA-allele:antigen combinations was detected, in four patients to two and in the other six patients only to one combination (Table 1). The fact that these endogenously processed HLA class I-restricted epitopes were recognized by CD8^+^ T cells present in the TIL population suggest that they all are involved in tumor control. In the end, a total of 20 CD8 T cell responses were detected in 16 HPV16-reactive T cell cultures, resulting in the identification of 11 different HLA-allele:epitope combinations.

**Table 1:** Summary of detected HPV16 E6 and E7-reactivities

Patient	HLA class I allele	Functional screen	Epitope recognized	Multimer staining	# of TCR with confirmed specificity	# of clones with tumor reactivity
C185	A*02:01	–	E7_11–19_	E7_11–19_	1	1
C446	B*07:02	–	E6_15–23_	E6_15–23_	1	1
H77	A*03:01	E7		n.t	n.d	–
	B*07:02	E7		n.t	n.d	–
H80	A*02:01	E6		n.t	–	–
	A*02:01	E7		n.t	–	–
H103	B*40:01	E7	E7_78–86_	n.t	n.d	–
H104	A*02:01	n.d	E6_29–38_	E6_29–38_	–	–
H133	A*02:01	E6	E6_29–38_	E6_29–38_	2	0
	B*07:02	E6	E6_15–23_	E6_15–23_	n.d	–
H159	A*02:01	n.d	E6_29–38_	E6_29–38_	–	–
H160	C*06:02	E6		n.t	n.d	–
	C*06:02	E7		n.t	n.d	–
H176	B*40:01	E7	E7_78–86_	n.t	4	3
H180	A*02:01	E6	E6_29–38_	E6_29-38_	–	–
	A*02:01	E7	E7_11–19_	E7_11-19_	–	–
	B*15:01	E7	E7_43–52_	n.t	1	1
H188	B*07:02	E6	E6_15–23_	E6_15–23_	–	–
	B*07:02	E6	E6_53–61_	n.t	1	0
	C*07:02	E6	E6_53–61_	n.t	3	2

*n.d.* not detected, *–* not tested

### Characterization of the identified HLA class I-restricted T cells

**Fig. 3:**
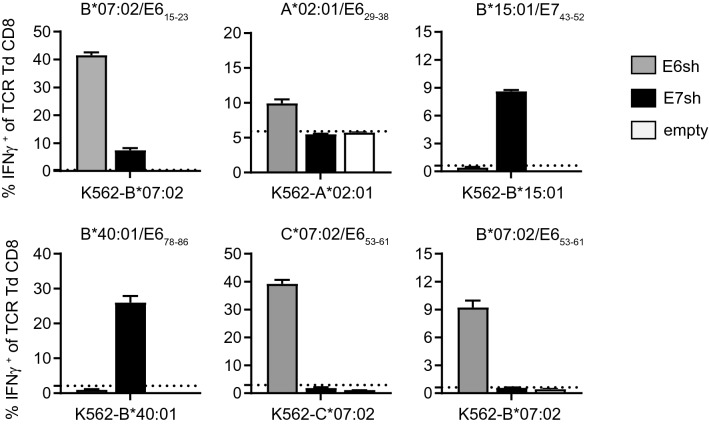
TCR transduced CD8^+^ T cells are capable of recognizing endogenously processed and presented epitopes on HLA class I allele and E6 or E7 engineered K562 cells. In vitro reactivity of TCR transduced (Td) CD8^+^ T cells against HLA class I and shuffled E6 (E6sh; gray), E7 (E7sh; black) or non-transduced (empty; white) engineered K562 cells was assessed by intracellular IFNγ staining and is depicted as percentage of IFNγ^+^ of TCR Td CD8^+^ T cells. Reactivity of TCR Td T cells alone is depicted by the dotted line. n.t. means not tested

**Fig. 4:**
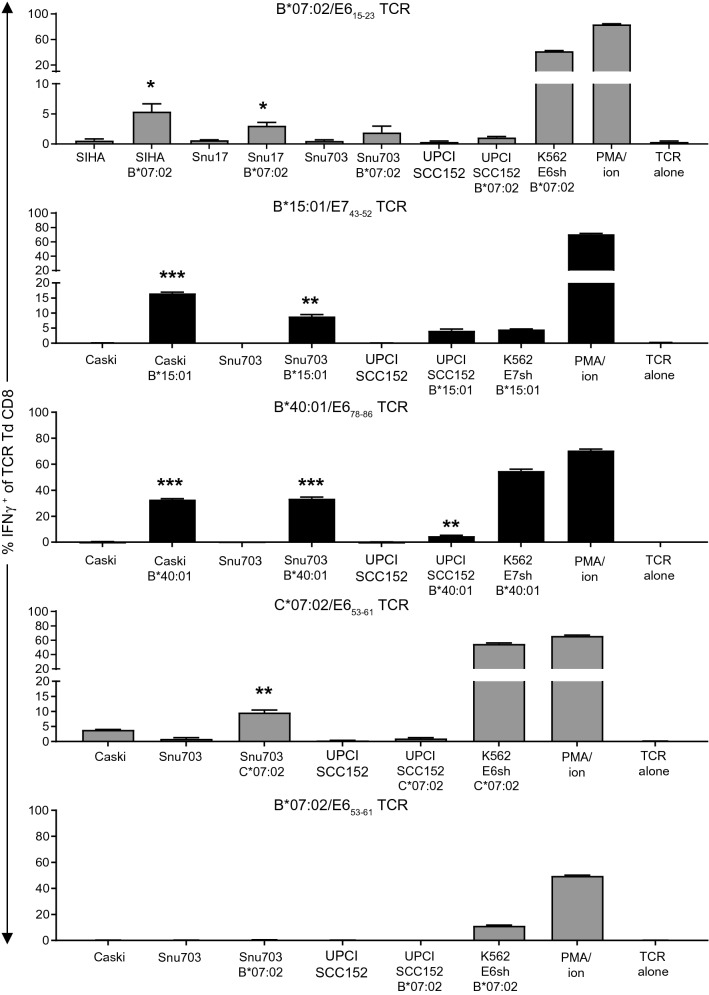
TCR transduced CD8^+^ T cells are capable of recognizing endogenously processed and presented epitopes on tumor cells. In vitro reactivity of TCR transduced (Td) CD8^+^ T cells against HPV16-expressing tumor cell lines that express the HLA class I allele of interest either naturally or after retroviral engineering (indicated underneath the cell line’s name) as assessed by intracellular IFNγ staining and depicted as percentage IFNγ^+^ of TCR Td CD8^+^ T cells. The percentage IFNγ^+^ of TCR Td T cells alone (TCR alone) is given as negative control and that in response to PMA and ionomycin (PMA/ion) or K562 engineered to express the HLA class I allele and E6sh or E7sh is given as positive control

In order to further characterize the identified epitopes and test their clinical relevance, the TIL were either directly stained with the specific HLA multimer (Fig. 2b) or with an IFNγ capture antibody following stimulation with K562 cells expressing the appropriate HLA class I molecule and E6 or E7 antigen (Fig. 2c), after which multimer^+^ or IFNγ^+^ CD8^+^ T cells were single cell sorted for direct TCR isolation and T cell cloning [25]. For seven HLA-allele:antigen combinations, one or more specific TCRs could be isolated and their functionality could be tested. Among the seven TCR were the previously described HLA-A*0201-restricted epitopes E6_29–38_ and E7_11–19_. Whereas the recognition of the naturally processed E7_11–19_ epitope in tumors by its specific TCR was excellent (Supplementary Figure 4), recognition of the E6_29–38_ epitope by its TCR was rather low, in spite of high peptide sensitivity of the TCR (Supplementary Figure 5), suggesting that this epitope may be poorly presented. The other five TCR, which were HLA-B*07:02, HLA-B*15:01, HLA-B*40:01 and HLA-C*07:02 restricted and recognize the E6_15–23_, E7_43–52,_ E7_78–86_ and E6_53–61_ epitopes respectively, displayed clear recognition of endogenously processed E6 or E7 antigen after transduction into primary human T cells (Fig. 3) Interestingly, five of the six TCRs with good recognition of endogenous E6 or E7 antigen in K562 cells also targeted naturally processed antigen in tumor cells. Although the recognition of the B*07:02-engineered tumor cell lines was not very high, the SIHA-B*07:02 and Snu17-B*07:02 cell lines were recognized by the B*07:02/E6_15–23_ TCR. These data indicate that these HLA class I restricted epitopes were processed and presented at a sufficient level (Fig. 4; Supplementary Figure 4) and thus are interesting candidates for adoptive cell transfer approaches to treat HPV16-induced cancers.

In order to understand whether the HLA class I-restricted CD8^+^ T cell epitopes clustered in a specific region of the HPV16 E6 and E7 oncoproteins or were confined to certain HLA class I alleles, we also performed a literature search for those CD8^+^ T cell epitopes that were recognized either by TIL or from other sources but then showed to recognize endogenously processed protein. This resulted in a list of 24 epitopes, including ours, which were spread evenly over the amino acid sequences of both E6 and E7 and presented by the HLA-A, -B, and -C class I alleles (Table 2).

**Table 2:** Summary of HLA class I processed and presented epitopes recognized by CD8^+^ T cells

Epitope	HLA restriction	Identification method	Level of evidence			Remark
			Endogenous recognition	Tumor recognition	Other	
E6_13–22_	B*07	TIL	n.t.	n.t.	APC + peptide	[4]
E6_15–23_	B*07:02	TIL	K562-shE6	SiHa, Snu17		This manuscript
E6_29–37_	B*48	PBMC of HPV-cleared women	VV-transfected APC	n.t.		[38]
E6_29–38_	A*02:01	TIL	K562-shE6,	No		This manuscript
		TIL, PBMC of HPV-cleared women	VV-transfected APC	CASKI, SSC90, SCC152		[4, 14, 38]
E6_31–38_	B*40:02	PBMC of HPV-cleared women	VV-transfected APC	n.t.		[38]
E6_52–61_	B*57:01, B*58, B*35	PBMC of HPV-cleared women or with an abnormal PAP smear, TIL	VV-transfected APC	n.t.	APC + peptide	[4, 39, 40]
E6_53–61_	B*07:02,	TIL	K562-shE6	No		This manuscript
E6_53–61_	C*07:02	TIL	K562-shE6	SNU703		This manuscript
E6_75–83_	B*62	PBMC of HPV-cleared women	VV-transfected APC	n.t.		[41]
E6_133–142_	B*68:01	PBMC of HPV-cleared women	VV-transfected APC	n.t.		[41]
E6_137–146_	B*27	TIL	n.t.	n.t.	APC + peptide	[4]
E6_149–158_	B*14	TIL	n.t.	n.t.	APC + peptide	[4]
E6	A*02:01	TIL	K562-shE6	n.t.		This manuscript
E6	C*06:02	TIL	K562-shE6	n.t.		This manuscript
E7_11–19_	A*02:01	TIL	K562-shE7	Caski, Snu17, SiHa, Snu703		This manuscript [4]
E7_11–20_	A*02:01	PBMC, TDLN and/or TIL;	VV-transfected APC	Caski		[6, 42, 43]
E7_43–52_	B*15:01	TIL	K562-shE7	SNU703, UPCI, SCC152, Caski		This manuscript
E7_61–69_	A*24:02	PBMC of HD after peptide stim.		SiHa		[17]
E7_67–76_	A*24:02	PBMC of HD after peptide stim.		SiHa		[17]
E7_78–86_	B*40:01	TIL	K562-shE7	Caski, SNU703		This manuscript
E7_86–93_	A*02:01	PBMC, TDLN and/or TIL;	VV-transfected APC	Caski		[6]
E7	A*02:01	TIL	K562-shE7	n.t.		This manuscript
E7	A*03:01	TIL	K562-shE7	n.t.		This manuscript
E7	B*07:02	TIL	K562-shE7	n.t.		This manuscript
E7	C*06:02	TIL	K562-shE7	n.t.		This manuscript

*HD* healthy donor, *nt* not tested, *sh* shuffled, *TDLN* tumor-draining lymph node, *TIL* tumor-infiltrating lymphocytes, *VV* vaccinia virus

## Discussion

Adoptive cell therapy is an attractive but highly personalized approach for the treatment of cancer and has been shown to work in patients with various forms of cancer [26–29]. The infused tumor-reactive T cells can respond to neoantigens [27, 30, 31], but in tumors of viral etiology they may also effectively target viral antigens [32, 33]. Here, a functional screening platform exploiting single HLA class I allele-engineered antigen presenting cells was applied to identify HPV16 E6 and E7 oncoprotein-derived epitopes that are recognized by CD8^+^ TIL and that were processed and presented in common HLA class I molecules. We identified six new epitopes and isolated TCRs to seven different HLA:epitope combinations, six of which displayed the capacity to recognize one or multiple HPV16^+^ tumor cell lines. The TCRs included one against the previously described clinically relevant HLA-A*02:01-restricted E7_11–19_ epitope [34] and one against the HLA-A*02:01-restricted E6_29–38_ epitope. For the latter epitope, the reactivity of the TCR to endogenous-processed peptide was rather low, but this may relate to the processing of this particular epitope as mass spectrometry data revealed good presentation of the E7 peptide but poor presentation of the E6 peptide [15, 19, 35]. Since HPV16 causes the majority of HPV-induced cancers and the identified epitopes are restricted by globally frequent HLA class I alleles [36, 37], the isolated TCRs may be a valuable tool for the treatment of many patients and thus function as an off-the-shelf product to produce tumor-specific T cells.

Although numerous HLA class I -restricted HPV16 E6 and E7 epitopes have been identified in the past decades by using various MHC-I immunogenicity prediction analysis and screening methodologies, there are only a few for which tumor presentation and T-cell recognition has been validated [6, 14, 17–19]. In most cases, the identification was based on the recognition of APC overexpressing the target antigens [6, 38–43]. Here we identified five new epitopes for which the isolated TCRs displayed recognition of endogenous E6 or E7 antigen when presented by tumor cells. The efficient detection of such novel tumor recognizing CD8^+^ T cells is most likely due to the use of the functional screening platform, which relies on the identification of T cells among TILs that respond to endogenously presented antigen on APC rather than on the identification of predicted epitopes. The latter is important as the accuracy of the computational models for HLA class I epitope prediction varies between the different alleles and is poor for rare or less-characterized MHC alleles [44, 45], while selection of putative epitopes relies on binding affinity decision thresholds [46]. This is also illustrated by our observation that only epitopes restricted to HLA-A*02:01 and HLA-B*07:02, alleles that are both well-characterized in terms of computational epitope screening methodologies, could be identified by the HLA class I multimer screening platform, whereas our functional screening platform identified epitopes restricted to the less well-characterized HLA-B*15:01, HLA-B*40:01 and HLA-C*07:02 alleles. For six cases we were unable to determine the epitope recognition because of a low sequence quality of the TCRs isolated.

In summary, we showed that our HLA class I functional screening platform could very efficiently identify novel HPV16 E6 and E7-restricted TCR that targeted naturally processed antigen in tumor cells in multiple MHC class I alleles. This opens possibilities to construct a broad HPV16 E6- and E7-specific TCR bank that could be used for off-the-shelf ACT treatment of patients with HPV16-induced cancers.

## Supplementary Information

Below is the link to the electronic supplementary material.

Supplementary file1 (PDF 485 kb)

## Data Availability

The data that support the findings of this study are available from the corresponding author upon reasonable request.

## Abbreviations

CBA: Cytometric bead array
CxCa: Cervical carcinomas
FFPE: Formalin-fixed paraffin-embedded
HPV16: Human papilloma virus type 16
IHC: Immunohistochemistry
MHC: Major histocompatibility complex
OPSCC: Oropharyngeal squamous cell carcinomas
TAP: Transporter associated with antigen processing
TCR: T cell receptor
TIL: Tumor infiltrating lymphocytes
