# Supplementary material for: The common HLA class I-restricted tumor-infiltrating T cell response in HPV16-induced cancer
Source: Cancer Immunol Immunother. 2022 Dec 16;72(6):1553–65. doi: 10.1007/s00262-022-03350-x (PMC10198845; doi:10.1007/s00262-022-03350-x)
Supplement: Supplementary file 1 — Supplementary file1 (PDF 485 kb) [file 262_2022_3350_MOESM1_ESM.pdf]

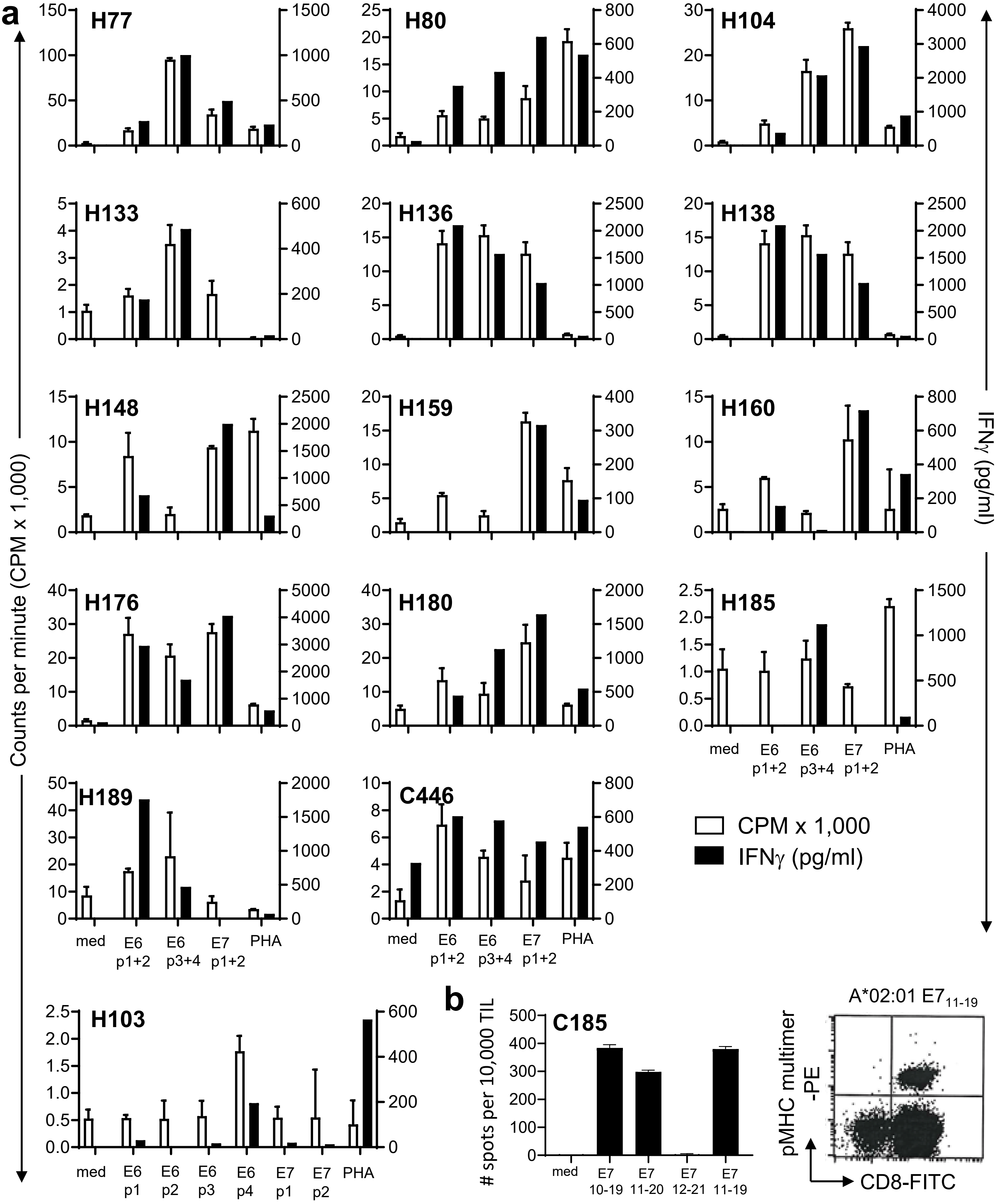

Supplementary figure 1. HPV16 E6 and E7 reactivity detected in TIL cultured from OPSCC and CxCa tumors. a) Cultured TIL of the indicated patients were tested against autologous HPV16 E6/E7 peptide (22-mers with 14 amino acids overlap)-loaded monocytes to determine the specificity of the T-cells using a combined proliferation/cytokine production assay. Proliferation is analyzed by 3H-Thymidine incorporation and given in counts per minute (CPM x 1,000; left Y-axis, open bars). IFNγ production was determined in supernatants of the proliferation assay and is given in pg/ml (right Y-axis; closed bars). **b)** Cultured TIL of CxCa patient C185 was tested against HPV16 E7 peptides 10-19, 11-20, 12-21 and 11-19 in an IFNγ ELISPOT assay (left) and stained with CD8 and HPV16 $E7_{11\text{-}19}$ pMHC multimer (right). Reactivity in the ELISPOTS is tested in triplicate, and the number of spots is given per 10,000 TIL.

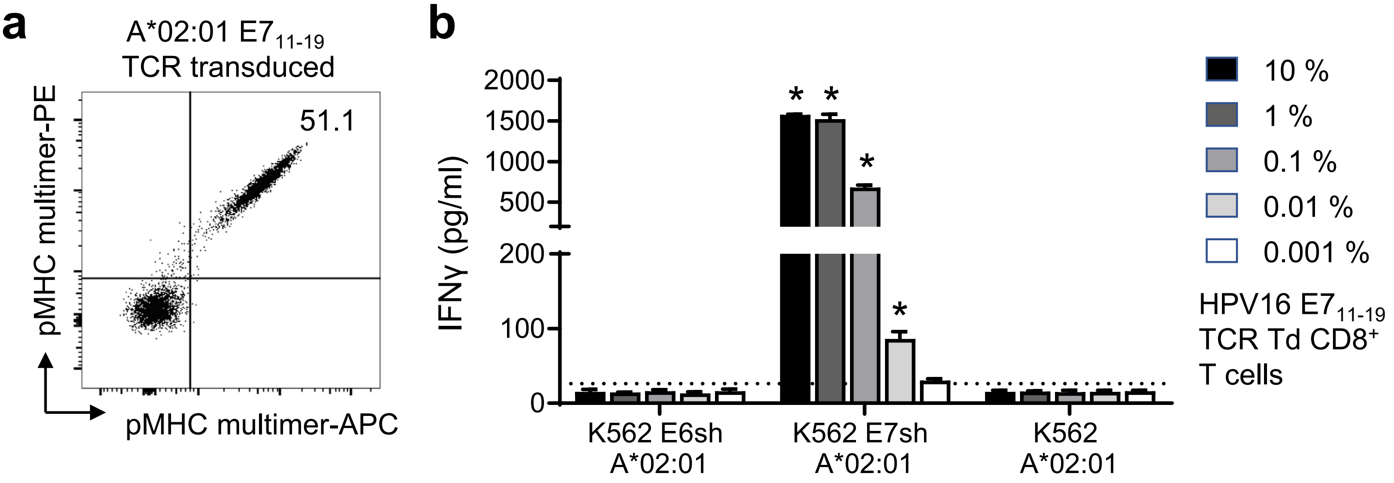

Supplementary figure 2. The HLA class I screening platform is a highly sensitive method to detect low frequency antigen-specific CD8⁺ T cells. a) pMHC multimer double staining of the HPV16 $E7_{11\text{-}19}$ TCR transduced (Td) primary T cells are depicted for a representative buffy coat. **b)** Graph depicting IFNγ production (in pg/ml) of E7-specific $CD8^+$ TCR Td T cells after overnight co-culture with K562 cells that have been transfected with shuffled HPV16 E6 DNA (E6sh), shuffled HPV16 E7 DNA (E7sh) and/or HLA-A*02:01. IFNγ is measured by cytometric bead array (CBA) and given for E7-specific TCR Td $CD8^+$ T cell lines harboring 10 (black), 1 (dark grey), 0.1 (grey), 0.01 (light grey) or 0.001 (white) percent of HPV16 $E7_{11\text{-}19}$ TCR Td $CD8^+$ T cells. Asterisk depicts the detection of a positive response, which is defined as IFNγ production that is at least two times that of TCR Td $CD8^+$ T cells alone (dotted line).

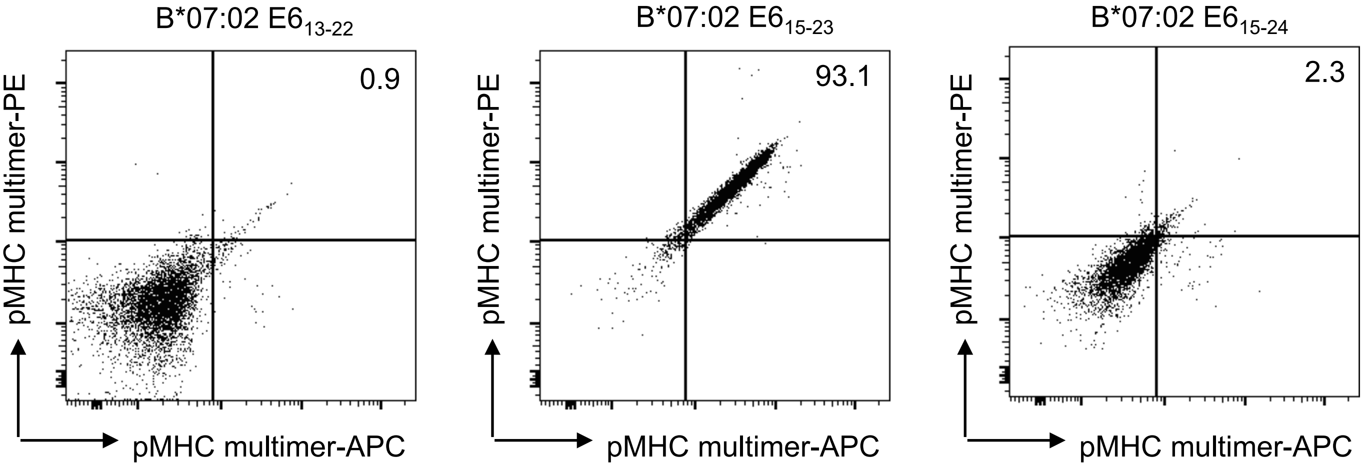

Supplementary figure 3. Epitope mapping using netMHC predicted epitopes allowed the identification of an HLA-B*07:02-restricted peptide $E6_{15-23}$. The C446 TIL-derived T cell line was stained with HLA-B*07:02 multimers loaded with HPV16 $E6_{13-22}$ (QERPRKLPQL), $E6_{15-23}$ (RPRKLPQLC) or $E6_{15-24}$ (RPRKLPQLCT) peptides and analyzed by flow cytometry.

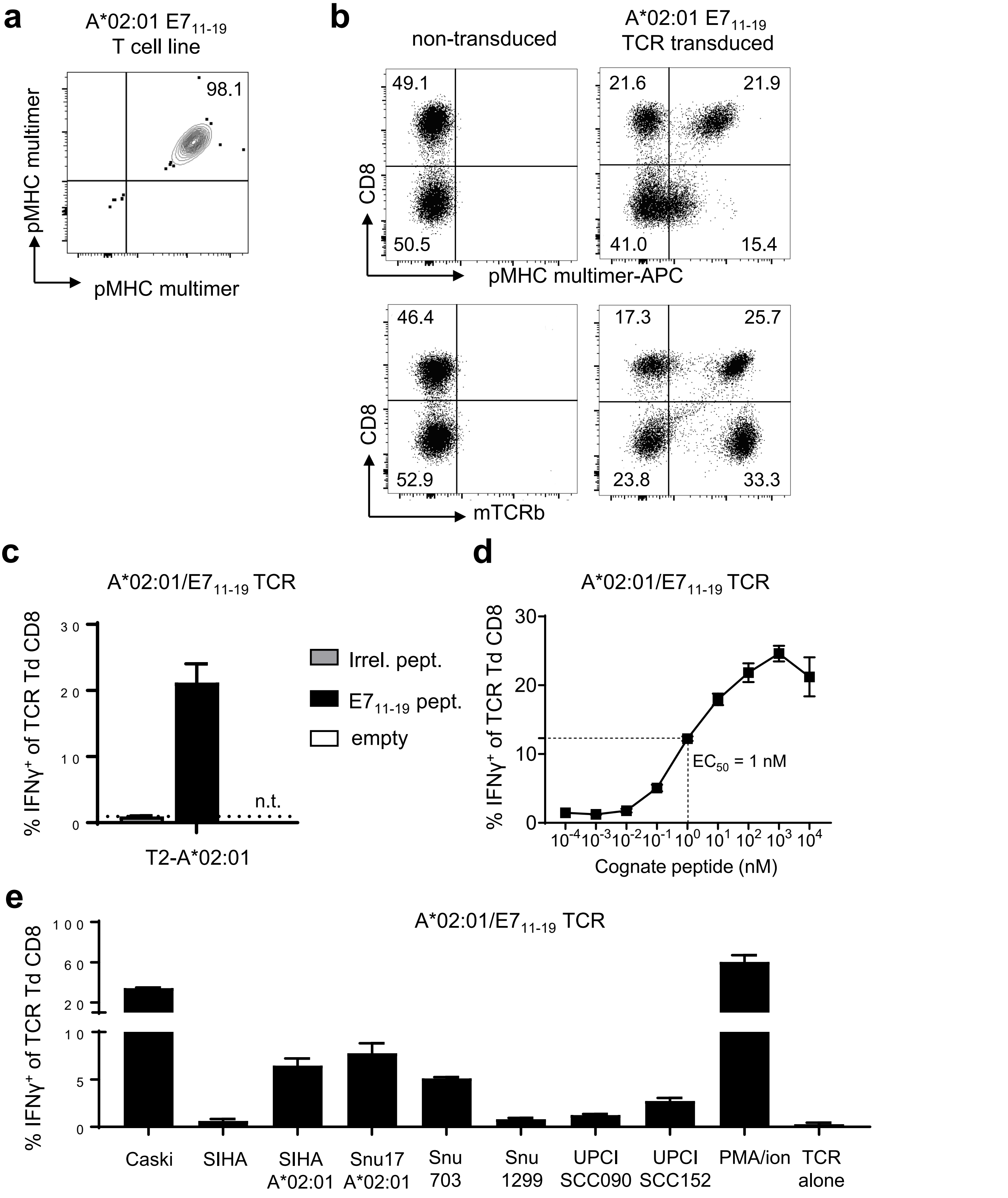

Supplementary Figure 4. Characterization of the C185-derived $E7_{11-19}$ HLA-A*02:01-restricted TCR. **a)** pMHC multimer staining of the C185 TIL-derived T cell line from which the TCR was isolated. **b)** Expression of the TCR following transduction into primary human T cells for a representative donor, as measured by pMHC multimer (top) and murine TCRβ constant domain antibody staining (bottom). **c)** In vitro reactivity of TCR transduced (Td) $CD8^+$ T cells against T2 cells loaded with irrelevant (grey) or $E7_{11-19}$ peptide (black), which was assessed by intracellular IFNγ staining and is depicted as percentage $IFN\gamma^+$ of TCR Td $CD8^+$ T cells. **d)** Assessment of the TCR sensitivity by peptide titration. **e)** In vitro tumor-reactivity of TCR Td $CD8^+$ T cells against HPV16-expressing tumor cell lines that express HLA-A*02:01 naturally or after retroviral engineering (indicated by A*02:01).

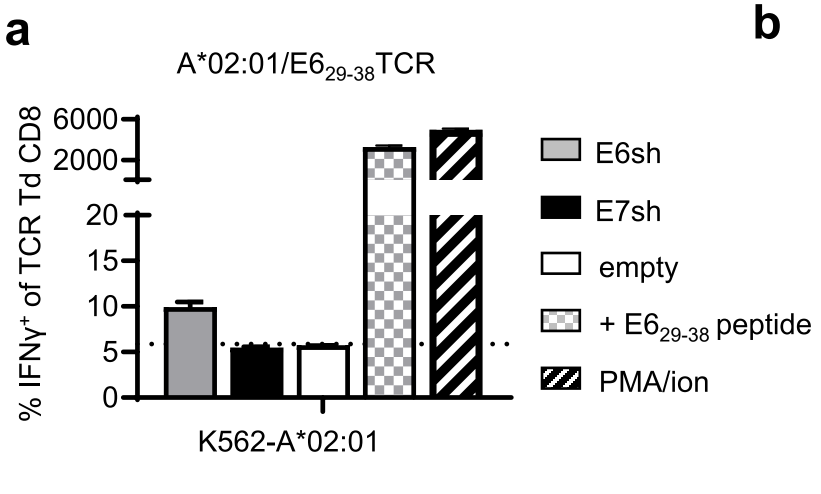

Supplementary figure 5. HPV16 $E6_{29\text{-}38}$ epitope demonstrated poor endogenous recognition. The H133 TIL-derived $E6_{29\text{-}38}$ epitope was tested against K562-A*02:01 cells that were transfected with E6sh (grey), E7sh (black), or were unloaded (empty) or loaded with 10 µM$E6_{29\text{-}38}$ peptide. PMA/ionomycin served as positive control (black diagonal striped). **a)** Graph depicting the percentage of $IFN\gamma^+$ cells of TCR Td $CD8^+$ T cells. Reactivity of TCR Td $CD8^+$ T cells alone is depicted by the dotted line. **b)** The TCR sensitivity is determined by peptide titration. The percentage of $IFN\gamma^+$ cells of TCR Td $CD8^+$ T cells in response to increasing concentration of cognate peptide is shown.

Supplementary Table 1. Patient characteristics

ID*	Tumor type	HLA-A$	HLA-A-2	HLA-B	HLA-B-2	HLA-C	HLA-C-2
C185	CxCa	**02:01**		**40:01**		03:04	
C446	CxCa	**01:01**	**03:01**	**07:02**	35:01	04:01	**07:02**
H77	OPSCC	**03:01**		**07:02**	**44:02**	**07:02**	
H80	OPSCC	**02:01**	**11:01**	**13:02**	**57:01**	**06:02**	
H103	OPSCC	**02:01**	**03:01**	39:01	**40:01**	**03:04**	**07:02**
H104	OPSCC	**02:01**	**03:01**	35:01	**51:01**	04:01	16:01
H133	OPSCC	**02:01**	68:02	**07:02**	14:02	**07:02**	08:02
H136	OPSCC	**01:01**	**24:02**	**51:01**	55:01	**01:02**	03:03
H138	OPSCC	30:01	31:01	**07:02**	**40:01**	**03:04**	**07:02**
H148	OPSCC	29:02		**44:03**		16:01	
H159	OPSCC	**02:01**	**24:02**	13:02	**15:01**	**01:02**	**06:02**
H160	OPSCC	**03:01**	**24:02**	18:01	37:01	**06:02**	**07:01**
H176	OPSCC	**02:01**	**03:01**	**35:01**	**40:01**	**03:04**	**04:01**
H180	OPSCC	**02:01**	**03:01**	**15:01**		**01:02**	02:02
H185	OPSCC	**02:01**	**11:01**	27:05	**40:01**	**01:02**	**03:04**
H188	OPSCC	**02:01**	**03:01**	**07:02**		**07:02**	

* "C" indicates Cervical Carcinoma (CxCa) patients included in the P08-197 CIRCLE study and "H" indicate OPSCC patients included in the P07-112 head and neck cancer study.

$ HLA type of interest in bold

Supplemental Table 2: Characteristics of the HPV16 E6 and E7 multimers

Class I allele	sequence	protein	Amino acid position
A*01:01	YSKISEYRHY	E6	77-86
	ISEYRHYCY	E6	80-88
	HGDTPTLHEY	E7	2-11
A*02:01	KLPQLCTEL	E6	18-26
	TIHDIILECV	E6	29-38
	FAFRDLCIV	E6	52-60
	TLEHEYMLDL	E7	7-15
	YMLDLQPET	E7	11-19
	YMLDLQPETT	E7	11-20
	TLEDLLMGTL	E7	78-87
	LLMGTLGIV	E7	82-90
	GTLGIVCPI	E7	85-93
A*03:01	IILECVYCK	E6	33-41
	CVYCKQQLLR	E6	37-47
	KFYSKISEY	E6	75-83
	KFYSKISEYR	E6	75-84
	KISEYRHYCY	E6	79-88
	TTLEQQYNK	E6	93-101
	LLIRCINCQK	E6	106-115
	CMSCCRSSR	E6	143-151
	GIVCPICSQK	E7	88-98
	IVCPICSQK	E7	89-98
A11*01:01	DIILECVYCK	E6	32-41
	IILECVYCK	E6	33-41
	CVYCKQQLLR	E6	37-47
	IVYRDGNPY	E6	59-67
	AVCDKCLKFY	E6	68-77
	KISEYRHYCY	E6	79-88
	TTLEQQYNK	E6	93-101
	GTTLEQQYNK	E6	94-101
	LLIRCINCQK	E6	106-115
	STLRLCVQSTH	E7	63-73
	GIVCPICSQK	E7	88-98
	IVCPICSQK	E7	89-98
A*24:02	MHQKRTAMF	E6	1-9
	VYDFAFRLD	E6	49-57
	VYDFAFRDLCI	E6	49-59
	PYAVCDKCL	E6	66-74
	PYAVCDKCLKF	E6	66-76
	EYRHYCYSL	E6	82-90
	HYCYSLYGTTL	E6	85-95
	CYSLYGTTL	E6	87-95
	QYNKPLCDL	E6	97-106
	QYNKPLCDLLI	E6	97-108
	CDSTLRLCV	E7	61-69
	LCVQSTHVDI	E7	67-76
	LMGTLGIVCPI	E7	83-93

B*07:02	QERPRKLPQL	E6	13-22
	RPRKLPQLC	E6	15-23
	RPRKLPQLCT	E6	15-24
	TPTLHEYMLDL	E7	5-15
B*08:01	LLRREVYDF	E6	44-52
	LLRREVYDFAF	E6	44-54
	ISEYRHYCYSL	E6	80-90
	EYRHYCYSL	E6	82-90
	LIRCINCQKPL	E6	107-117
	DKKQRFHNI	E6	127-135
	NIRGRWTGRCM	E6	134-144
	SSRTRRETQL	E6	149-158
C*07:02	LLRREVYDFAF	E6	44-54
	LRREVYDFAF	E6	45-54
	LRREVYDFAFR	E6	45-55
	RREVYDFAF	E6	46-54
	RREVYDFAFR	E6	46-55
	YRDGNPYAV	E6	61-69
	KFYSKISEY	E6	75-83
	SEYRHYCYSL	E6	81-90
	EYRHYCYSL	E6	82-90
	YRHYCYSLY	E6	83-91
	CYSLYGTTL	E6	87-95
	QYNKPLCDL	E6	98-106
	SRTRRETQL	E6	150-158
	TLHEYMLDL	E7	7-15
	RAHYNIVTF	E7	49-57
	HYNIVTFCC	E7	51-59
	FCCKCDSTL	E7	57-65
	LRLCVQSTH	E7	65-73

Supplemental Table 3. Patient tested HLA alleles and identified CD8⁺ T cell epitopes

HLA	C185	C446	H77	H80	H103	H104	H133	H136	H138	H148	H159	H160	H176	H180	H185	H188
A*01:01								MS/FS								
A*02:01	$E7_{11-19}$			E6<br>E7	MS<br>FS	$E6_{29-38}$<br>FS	$E6_{29-38}$				$E6_{29-38}$<br>FS		MS	$E6_{29-38}$<br>$E7_{11-19}$	FS	FS
A*03:01			E7		MS/FS	MS/FS						FS	MS	MS		MS/FS
A*11:01				MS/FS											FS	
A*24:02								MS/FS			MS/FS	FS				
B*07:02		$E6_{15-23}$	E7				$E6_{15-23}$		MS							$E6_{15-23}$<br>$E6_{53-61}$
B*08:01																
B*13:02				FS												
B*15:01											FS			$E7_{43-52}$		
B*35:01													FS			
B*40:01					$E7_{78-86}$				FS				$E7_{78-86}$		FS	
B*44:02			FS													
B*44:03										FS						
B*51:01						FS		FS						FS		
B*57:01				FS												
C*01:02								FS			FS			FS	FS	
C*02:02																
C*03:04					FS				FS				FS		FS	
C*04:01						FS							FS			
C*05:01																
C*06:02				FS							FS	E6, E7				
C*07:01												FS				
C*07:02			FS		MS/FS		MS/FS		MS/FS							$E6_{53-61}$

Light grey indicates CD8⁺ T cell recognition by pMHC class I multimer detection, dark grey indicates recognition by functional screen and black indicates recognition by both multimer and functional screen for the indicated epitope when provided. White shows the HLA alleles that remained untested (empty) or were tested with multimer screen (MS) or functional screen (FS) but showed no detectable T cell responses.
